# Editorial: Women in lens and cataract

**DOI:** 10.3389/fopht.2025.1616466

**Published:** 2025-05-09

**Authors:** Catherine Cheng, Barbara Pierscionek

**Affiliations:** ^1^ School of Optometry and Vision Science Program, Indiana University, Bloomington, IN, United States; ^2^ Faculty of Health Medicine and Social Care, Medical Technology Research Centre, Anglia Ruskin University, Chelmsford, United Kingdom

**Keywords:** lens, cataract, biomechanics, oxidation, microcirculation, signalling, glutathione, connexin

The ocular lens is a highly organized and complex tissue that is an ideal model for understanding the challenges of aging and long-term cellular and molecular homeostasis. This transparent and ellipsoid tissue in the anterior chamber of the eye changes shape to fine-tune the focusing of light onto the retina to form a clear image. The lens is suspended behind the iris by a ring of thin zonules that are connected to the ciliary muscles. The tension exerted by the zonules on the lens changes during muscle contractions that allow for the lens shape changes to focus on near objects through the process of accommodation.

At first glance, the lens is deceptively simple because it is devoid of blood vessels and nerves, and the entire tissue is made up of just two cell types, a monolayer of epithelial cells covering the anterior hemisphere and a bulk mass of fiber cells that differentiate from epithelial cells. Yet, the seeming lack of complexity in structure and function is enigmatic; the structure/function relationship between lens proteins and its optical and biomechanical properties, how the lens proteins change with age, and what mechanisms lead to age-related pathologies remain unanswered. The lens poses extraordinary challenges for cellular homeostasis because all the lens cells ever made in the lens are retained in the tissue. The lens never loses or sheds any cells, and it continues to grow by accruing new layers of cells overlaid onto existing generations of lens fiber cells, akin to the growth mode that creates rings in a tree. The oldest lens cells, which are also some of the oldest cells in the body, are at the center of tissue through which the optic axis passes. As an avascular tissue, the lens generates its own microcirculation current to transport of nutrients, antioxidants, and other small molecules to maintain life-long growth and homeostasis.

The elegance of this clear and light-focusing tissue is only revealed at close examination of the cell-cell interactions and 3D architecture. The review by Greiling et al. undertakes an investigation of the lens structure/function relationship from a different perspective: that of the lamellar formation of the lens that is a consequence of its mode of growth and how this may be linked to its microcirculation and hence maintenance of transparency. Work by Crews et al. considers the biomechanical aspects and how changes in lens shape and tension may impact on transport of nutrients. Variability of diffusion patterns between anterior and posterior lens poles was noted for three of the dyes, and one dye suggested an active transport mechanism may be present. The lens microcirculation pathway requires large gap junction plaques made from connexin proteins, and the importance of gap junction communication in lens and eye growth and homeostasis are revealed in two articles. Painter et al. used optical coherence tomography to reveal that loss of connexin 50 in mouse lenses led to changes in anterior chamber depth, suggesting that normal lens size and growth are a prerequisite for normal eye size and development. Sellitto and White show that connexin 50, hosphoinositide 3-kinase (PI3K), and phosphatase and tensin homolog (PTEN) all play roles in lens size and growth.

The prevention or minimization of protein oxidation is another factor for maintaining lens homeostasis. A fascinating study on circadian rhythms in the lens presents a number of avenues for further study on regulation of a major antioxidant, namely glutathione (Li et al.). The implications of these results are that glutathione and enzymes responsible for its homeostasis may be dependent on circadian rhythms and that oxidative stress in the lens could be regulated by manipulating the circadian clock. This offers a novel and exciting means of controlling the health of the lens and potentially maintaining transparency.

Cataracts, defined as any opacity in the lens, remain the leading cause of blindness in the world. We continue to explore the genes, signaling pathways, age-related protein changes, and external factors that can lead to cataractogenesis. Choquet et al. reveal novel genetic markers for age-related cataract development, suggesting markers for genetic susceptibility to cataracts may exist outside the lens tissue. Dysfunction of the Eph/ephrin bidirectional signaling pathway has been linked to congenital and age-related cataracts in human patients. Huynh and Cheng conducted a thorough investigation of the expression levels of the Eph/ephrin genes in the lens that change with age or due to disruption of the signaling pathway. A detailed mass spectrometry study by Paredes et al. describes the irreversible crosslinking of proteins through the formation of reactive intermediates, dehydroalanine (DHA) and dehydrobutyrine (DHB). Increased levels of DHA and DHB are found in cataractous lenses, suggesting that protein crosslinking is linked to age-related opacities. The role of light, and in particular UV light, on the biochemical properties of the lens is reviewed by MacFarlane et al. Whether UV exposure leads to cataract or expediates the process of cataract formation has been debated for decades. MacFarlane et al. considered several different animal models of UV light exposure to gain a deeper comprehension of the effects of UV light on lens structure and function. Collectively, these studies reveal the potential causes for cataracts from genetic variation to protein aggregation to exposure to UV rays. It is likely that a combination of these factors contributes to the development of cataracts. Recent focus on elucidating the mechanisms for cataractogenesis, especially age-related opacities, will lead to novel non-surgical methods to prevent or delay cataracts.

We were delighted to curate this special topic with 7 original articles and 2 reviews to highlight the contribution of women to vision science and lens research. While there has been an increase in women in STEM, we welcome further opportunities to foster scientific curiosity in girls and encourage new generations of women to pursue careers in engineering, math, and science.

